# Multicomponent X-ray Shielding Using Sulfated Cerium Oxide and Bismuth Halide Composites

**DOI:** 10.3390/molecules28166045

**Published:** 2023-08-14

**Authors:** Shanmugam Mahalingam, Dae-Seong Kwon, Seok-Gyu Kang, Junghwan Kim

**Affiliations:** 1 Department of Materials System Engineering, Pukyong National University, Busan 48513, Republic of Korea; shanmugam82@pknu.ac.kr (S.M.); eotjd2065@pukyong.ac.kr (D.-S.K.); rkdtjrrb714@pukyong.ac.kr (S.-G.K.); 2 Institute of Energy Transport and Fusion Research, Pukyong National University, Busan 48513, Republic of Korea

**Keywords:** sulfated, cerium oxides, porous matrix, bismuth halides, X-ray shielding

## Abstract

Lead is the most widely used X-ray-shielding material, but it is heavy (density ≈ 11.34 g/cm^3^) and toxic. Therefore, the replacement of Pb with lightweight, ecofriendly materials would be beneficial, and such materials would have applications in medicine, electronics, and aerospace engineering. However, the shielding ability of Pb-free materials is significantly lower than that of Pb itself. To maximize the radiation attenuation of non-Pb-based shielding materials, a high-attenuation cross-section, normal to the incoming X-ray direction, must be achieved. In this study, we developed efficient X-ray-shielding materials composed of sulfated cerium oxide (S-CeO_2_) and bismuth halides. Crucially, the materials are lightweight and mechanically flexible because of the absence of heavy metals (for example, Pb and W). Further, by pre-forming the doped metal oxide as a porous sponge matrix, and then incorporating the bismuth halides into the porous matrix, uniform, compact, and intimate composites with a high-attenuation cross-section were achieved. Owing to the synergetic effect of the doped metal oxide and bismuth halides, the resultant thin (approximately 3 mm) and lightweight (0.85 g·cm^−3^) composite achieved an excellent X-ray-shielding rate of approximately 92% at 60 kV, one of the highest values reported for non-heavy-metal shielding materials.

## 1. Introduction

Ionizing radiation, including γ-rays, X-rays, and neutrons, is widely utilized in the nuclear, military, space, and medical fields [1,2]. In particular, X-rays are the most frequently utilized radiation in medical diagnosis, therapy, industrial inspection, and academic research [3,4,5]. Therefore, the demand for X-ray-based technology is expected to increase continuously. However, excessive exposure to X-rays is harmful. In particular, X-ray radiation exposure, particularly long-term high-energy radiation exposure, can cause cancer [6,7]. Therefore, the use of X-rays as an analytical tool presents a risk for the instrument operators [8,9]. Consequently, adequate X-ray protection is crucial to keep workers safe, and numerous strategies for developing effective X-ray radiation-shielding materials have been proposed [10,11,12,13].

Owing to its high density and *Z* value, lead is the most effective material for radiation shielding, particularly for preventing γ-ray and X-ray penetration [14]. Typical Pb-based shielding materials comprise Pb particles impregnated with Si or rubber [15,16]. However, a disadvantage of Pb-based materials is their toxicity, and their potential for leakage as a result of matrix damage, cracking, and aging [15]. Additionally, the majority of Pb-based shielding materials are heavy and bulky, and their applications in wearable radiation-protective clothing are hampered by their lack of flexibility and weight [17]. Therefore, for convenience and practicality, lightweight and ecofriendly non-Pb shielding materials are required [18].

Metals (e.g., W, Ba, and Sn) and metal oxides (e.g., WO_3_, CuO, and Bi_2_O_3_) of high-Z elements have been studied for use in non-Pb-based radiation-shielding materials, to solve these issues [19,20,21]. Because metals and metal oxides are usually processed in powder form, polymers are frequently mixed together as supporting components. Using these composites, a wide range of desired shapes can be obtained, including plates, fibers, and sheets. However, the majority of X-ray-shielding composites described in the literature contain more than 50% polymer, which makes only a small contribution to the X-ray attenuation [21,22,23]. Consequently, the shielding performance is dependent on the distribution of metal particles in the polymer matrix, and is limited by the polymer components in the composites.

To achieve a high radiation attenuation, the interaction between the radiation and the shielding material must be maximized; that is, the attenuation cross-section must be high. This is directly affected by the particle dispersion in the composites. However, because the dispersion of metal particles is sensitive to many factors, such as the miscibility, temperature, and humidity, achieving a uniform and reproducible composite remains a challenge. Another factor for efficient radiation shielding is a high electron density in the materials. High-Z elements have high electron densities, but they are usually heavy, owing to their higher proton and neutron numbers. Therefore, doping is a promising strategy to increase the electron density of materials without increasing their weight.

In this study, novel multicomponent X-ray-shielding materials composed of sulfated cerium oxide (S-CeO_2_) and bismuth halides were developed for the first time. As the Ce (ρ: 6.76 g/cm^3^, Z: 58) and Bi (ρ: 9.78 g/cm^3^, Z: 83) are relatively low-density metals with high Z values, their derivatives of CeO_2_ and bismuth halides were explored for X-ray shielding in this work. Both S-CeO_2_ and bismuth halides exhibit good X-ray attenuation abilities. Owing to the successful surface sulfation of CeO_2_ crystals (sulfate ratio of approximately 6.5%), the S-CeO_2_ showed enhanced shielding ratios compared to pristine CeO_2_. In addition, we pre-formed the S-CeO_2_ in a porous polymer-based sponge matrix, and then incorporated bismuth halides into the porous matrix; this strategy was used because the bismuth halides would otherwise be immiscible. The resultant materials were uniform, compact, and intimate composites, with a high-attenuation cross-section. After optimization, the multicomponent X-ray shielding exhibited an excellent X-ray-shielding rate of approximately 92% at 60 kV, which is among the highest values reported for non-heavy-metal shielding materials. Furthermore, the composites are lightweight (0.85 g·cm^−3^), mechanically flexible, and ecofriendly, because of the absence of Pb. We expect that this study will pave the way for the development of efficient and lightweight radiation-shielding materials, using a combination of doped metal oxides and metal salts.

## 2. Results and Discussion

**Figure 1:**
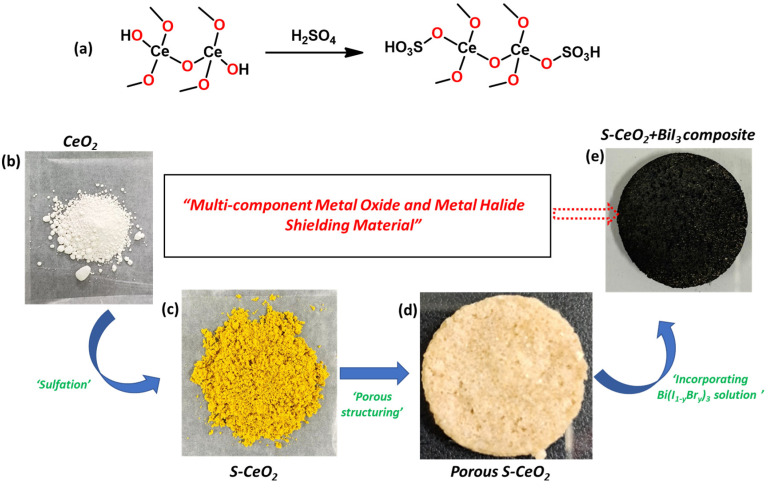
Preparation of the S-CeO_2_ nano powder and S-CeO_2_/BiI_3_ composite shielding material. (**a**) Scheme of sulfonation of CeO_2_. Photographs of (**b**) pure CeO_2_, (**c**) S-CeO_2_, (**d**) porous S-CeO_2_ coin-shaped sponge, and (**e**) S-CeO_2_/BiI_3_ composite.

Figure 1 shows the fabrication of the S-CeO_2_/BiI_3_ composite, as well as photographs of each product. There were three fabrication steps: (1)sulfation, (2) porous structuring, and (3) the incorporation of bismuth halides. First, CeO_2_ was sulfated by chemically bonding sulfonic (–SO_3_H) groups to the CeO_2_ surface, as shown in Figure 1a. Sulfation was confirmed by the color change from white (CeO_2_) to yellow (S-CeO_2_) powder, as shown in Figure 1b,c. Sulfation is a good strategy for increasing the surface electron density of CeO_2_, thereby improving radiation shielding [24]. Sulfation also increases the catalytic activity and stability of the metal oxides [25,26,27]. Subsequently, a porous S-CeO_2_ sponge was fabricated as an X-ray-shielding material (Figure 1e). Crucially, for shielding applications, the metal oxide powders should be moldable, enabling the formation of shapes, such as plates, fibers, or films. To achieve this, polymers (for example, epoxy or PDMS) are required as binders. However, because the polymers make a very small contribution to the X-ray attenuation, we introduced a third component with good X-ray attenuation. This was achieved by pre-forming a porous S-CeO_2_ sponge, and then soaking it in a bismuth halide solution (Figure 1d,e). As a result, a multicomponent X-ray-shielding sponge containing S-CeO_2_ and bismuth halide was formed, as shown in Figure 1e. Because both S-CeO_2_ and bismuth halides exhibit a good X-ray attenuation, their combination is expected to result in efficient X-ray shielding.

### 2.1. Structural and Functional Group Analysis of S-CeO_2_

**Figure 2:**
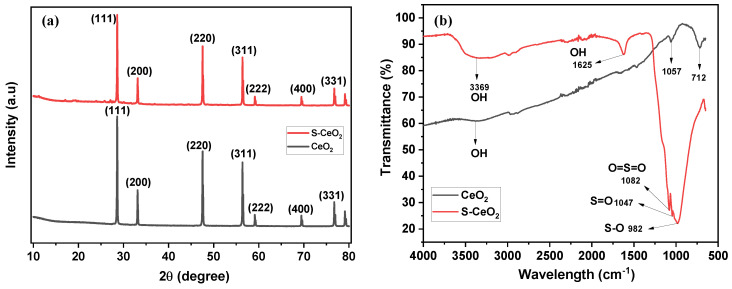
(**a**) Powder XRD patterns and (**b**) FT-IR spectra of pure CeO_2_ (black) and S-CeO_2_ (red).

The powder XRD (PXRD) patterns of pure CeO_2_ and S-CeO_2_ are shown in Figure 2a. The diffraction patterns of CeO_2_ and S-CeO_2_ contain sharp and intense peaks, confirming the good crystallinity of these materials. The peaks were indexed to JCPDS Standard No. 65-5923 for CeO_2_, and the characteristic peaks in the PXRD pattern of CeO_2_ (before sulfation) were detected at 28.56° (111), 33.12° (200), 47.59° (220), 56.39° (311), 59.14° (222), 69.52° (400), and 76.86° (331) in 2*θ*, consistent with the cubic fluorite structure of CeO_2_. The PXRD pattern of S-CeO_2_ was identical to the standard card, indicating that the introduction of sulfur had no effect on the crystallinity. The sulfation process may prevent the CeO_2_ particles from clumping together, which could increase their surface area, and reduce their crystalline size. This is consistent with the observed decrease in crystallite size. However, the X-ray diffraction patterns did not show any significant peak shifts, suggesting that sulfation did not affect the crystal structure or phase composition of CeO_2_.

Figure 2b shows the FT-IR spectra of pure CeO_2_ and S-CeO_2_. In the spectrum of pure CeO_2_, the O-H stretching, CO_2_ asymmetric stretching, and C-O stretching vibrations were observed at 3369, 712, and 1057 cm^−1^, respectively. Figure 2b shows the FT-IR spectrum of S-CeO_2_. The bands observed for S-CeO_2_ indicate that its structure is substantially different from that of pure CeO_2_. The peak observed at 1625 cm^−1^ corresponds to the O-H stretching vibrations of the O-H group on the surface of S-CeO_2_ [28]. The observed peaks at 1087, 1047, and 982 cm^−1^ correspond to the stretching of the O=S=O, S=O, and S-O groups, respectively, in the sulfonic acid group of the S-CeO_2_ nanostructure. In addition, the sulfonic acid group (-SO_3_H) yielded a peak at around 3369 cm^−1^, which is similar to the stretching vibration of water molecules (-OH) [28]. These results confirm that the sulfation reaction introduced sulfonic acid groups onto the surface of the CeO_2_ nanostructure.

### 2.2. XPS Analysis of S-CeO_2_

**Figure 3:**
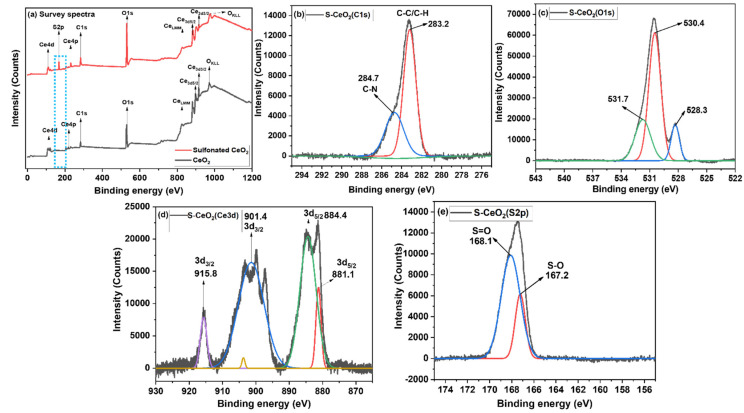
XPS (**a**) survey spectra of CeO_2_ and S-CeO_2_ (**b**), C1s, (**c**) O1s, and (**d**) Ce3d core-level spectra and (**e**) S2p spectrum of S-CeO_2_.

The XPS profiles of CeO_2_ and S-CeO_2_ are shown in Figure 3. Figure 3a shows the survey spectra of CeO_2_ and S-CeO_2_, and peaks corresponding to all the expected elements were observed. However, a new peak in the S-CeO_2_ spectrum with a binding energy of 167 eV was also observed (blue dashed frame), and this can be attributed to the S2p component, demonstrating that sulfonic acid groups were bound to CeO_2_. Figure 3b shows the C1s spectrum of S-CeO_2_, and the peaks were deconvoluted into two peaks at 284.7 and 283.2 eV, which correspond to C-N and C-C/C-H, respectively. As shown in Figure 3c, the deconvolution of the O1s electron core-level spectrum revealed three distinct oxygen species. The high-intensity peak at 530.4 eV is associated with oxygen in the CeO_2_ lattice [29]. In contrast, the other two peaks at 531.7 and 528.3 eV may be due to adsorbed oxygen or hydroxyl groups present in the oxygen vacancy sites within the CeO_2_ matrix [30,31]. The high-resolution Ce3d spectrum (Figure 3d) reveals four peaks that can be attributed to the spin–orbit splitting of Ce 3d_5/2_ and Ce 3d_3/2_, respectively [32]. Because of this spin doublet splitting, CeO_2_ can be found in both the Ce^3+^ and Ce^4+^ oxidation states [33]. The distinctive XPS signals at 884.4 and 901.4 eV can be assigned to the Ce^4+^ 3d_5/2_ and Ce^3+^ 3d_3/2_ electron states, respectively. The two additional satellite peaks observed at 881.1 and 915.8 eV originate from Ce^3+^ 3d_5/2_ and Ce^4+^ 3d_3/2_, respectively. Thus, the recorded spectra confirm the presence of the mixed valences Ce^3+^ and Ce^4+^. Further, the primary peaks at 884 and 901 eV indicate the relative quantities of Ce^4+^ and Ce^3+^ in the sample. The presence of oxygen vacancies in CeO_2_ was confirmed using the areas of the individual peaks, which reveal that the concentration of Ce^4+^ was relatively high. It was determined that these two states have a binding energy difference of 17 eV, which is in good agreement with accepted values [34]. In addition, sulfate groups were detected on the CeO_2_ surface, as evidenced by the peaks at 168.1 and 167.2 eV for S 2p_1/2_ and 2p_3/2_ in the S2p spectra of S-CeO_2_ (Figure 3e), which were assigned to S=O and S-O, respectively [26].

### 2.3. HR-TEM and Element Mapping Analysis of S-CeO_2_

**Figure 4:**
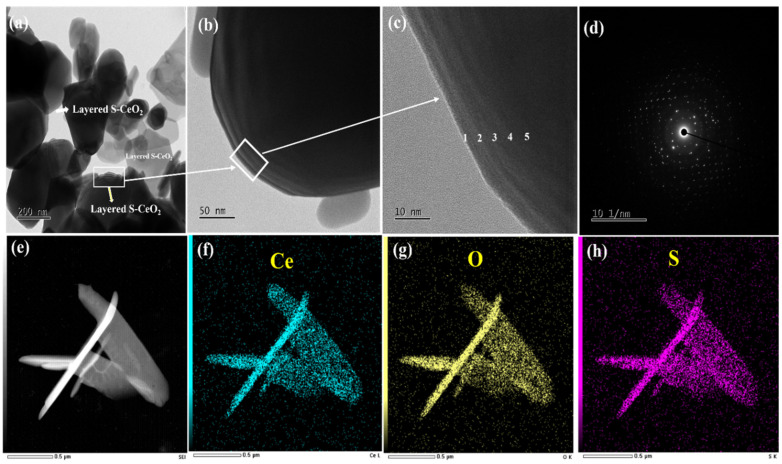
HR-TEM images of S-CeO_2_ (**a**–**c**) different magnifications, and (**d**) SAED patterns of S-CeO_2_. (**e**) HAADF-STEM image (**f**) and element maps of Ce, (**g**) O, and (**h**) S.

Additionally, HR-TEM was used to study the surface morphology of sulfated CeO_2_, as shown in Figure 4. Figure 4a–c display the HR-TEM images of S-CeO_2_ at various magnifications. As shown in Figure 4a, the S-CeO_2_ crystals with diameters of 100–200 nm were highly dispersed. In addition, the S-CeO_2_ crystals have a thin and transparent semi-hexagonal plate-like structure, and stacked layers can be seen at the edges. The marked region indicates the thin, layered structure of S-CeO_2_, which may be helpful for the surface modification of CeO_2_ crystal with sulfonic acid groups, thereby enhancing the electron density and X-ray-shielding activity. Figure 4b,c show the layered structure of the S-CeO_2_ crystals more clearly. Notably, there is a five-layer structure at the edge of the S-CeO_2_ crystals (Figure 4c). The SAED pattern of the S-CeO_2_ crystal structure is shown in Figure 4d, illustrating the highly crystalline nature of S-CeO_2_. The reflections are consistent with the cubic fluorite structure. Field-emission electron probe microanalysis (EPMA) was also performed on S-CeO_2_, and element maps were recorded (Figure S2). Figure 4e–h illustrate the S-CeO_2_ EPMA maps and the associated EDX data. As shown, cerium, oxygen, and sulfur atoms were uniformly distributed throughout the components. In particular, the sulfur distribution in Figure 4h clearly shows that the sulfation was successful, which is consistent with the FT-IR and XPS results. The EDX spectrum presented in Figure S2 also confirms the elemental composition of the prepared S-CeO_2_.

### 2.4. FE-SEM and Element Mapping

**Figure 5:**
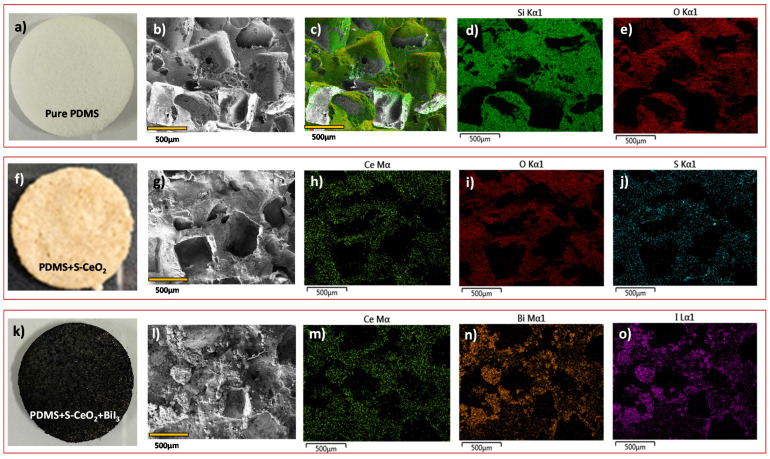
Photographs and element maps of PDMS, PDMS with S-CeO_2,_ and PDMS/S-CeO_2_/BiI_3_. (**a**) Photograph of the pure PDMS. (**b**–**e**) HAADF image and S/O overlapped element map and individual S and O maps of the pure PDMS. (**f**) Photograph of PDMS with S-CeO_2_. (**g**–**j**) HAADF image and element maps for Ce, O, and S of PDMS with S-CeO_2_. (**k**) Photograph of PDMS/S-CeO_2_/BiI_3_. (**l**–**o**) HAADF image and element maps for Ce, Bi, and I of PDMS/S-CeO_2_/BiI_3_. The scale bars are 500 µm in all HAADF images.

We fabricated a sponge-type composite for shielding applications, using S-CeO_2_ nanopowder and bismuth halides as X-ray attenuators, with PDMS as a binder (Figure 5k). We also prepared pure PDMS and PDMS/S-CeO_2_ sponges for comparison (Figure 5a,f). We used FE-SEM and EDX elemental mapping to examine the morphologies and elemental distributions of the pure porous PDMS, PDMS/S-CeO_2_, and PDMS/SCeO_2_/BiI3. The FE-SEM images of the pure porous PDMS (Figure 5b and Figure S3) reveal a porous structure with interconnected channels, which could facilitate the incorporation of S-CeO_2_ and bismuth halides. Elemental mapping confirmed that the pure PDMS contained Si and O, and they were evenly distributed throughout the structure (Figure 5c–e). The EDX data in Figure S4 further confirm the elemental composition and purity of the pure PDMS. The surface morphology of the PDMS/S-CeO_2_ sponge is shown in Figure 5g. As shown, it has a highly porous and spongy structure. The high-magnification image in Figure S5 clearly shows that nanostructured S-CeO_2_ is uniformly distributed throughout the PDMS matrix, and retains its porous and spongy structure. Elemental mapping confirmed that S-CeO_2_ was uniformly distributed, and formed the main matrix in the composite (Figure 5h–j). The EDX data in Figure S6 further verify the elemental composition of the PDMS/S-CeO_2_. During our preparation process, the PDMS/S-CeO_2_/BiI_3_ was prepared through the soaking of the PDMS/S-CeO_2_ sponge in a bismuth halide solution (BiI_3_ + BiBr_3_). Thus, the bismuth halide salts penetrated the sponge and solidified on the surfaces of the pores of the PDMS/S-CeO_2_ sponge. This was reflected by a color change from light-yellow PDMS/S-CeO_2_ to black PDMS/S-CeO_2_/BiI_3_ (Figure 5k). The SEM image of PDMS/S-CeO_2_/BiI_3_ also reveals that bismuth halide particles are uniformly dispersed throughout the PDMS matrix (Figure 5l and Figure S7). Thus, the S-CeO_2_ acted as a scaffold for the BiI_3_ particles, allowing them to be uniformly dispersed throughout the PDMS matrix. The combination of these three materials results in a composite with unique properties. Figure 5m–o show the elemental maps of the PDMS/S-CeO_2_/BiI_3_ sponges. The elemental maps and EDX spectrum in Figure S8 also reveal a homogeneous distribution of Ce, Bi, and I. The quantitative data on the chemical composition of the PDMS/S-CeO_2_/BiI_3_ shielding material are also shown in Table S1.

### 2.5. X-ray-Shielding Analysis of Multicomponent Halide Composites

**Figure 6:**
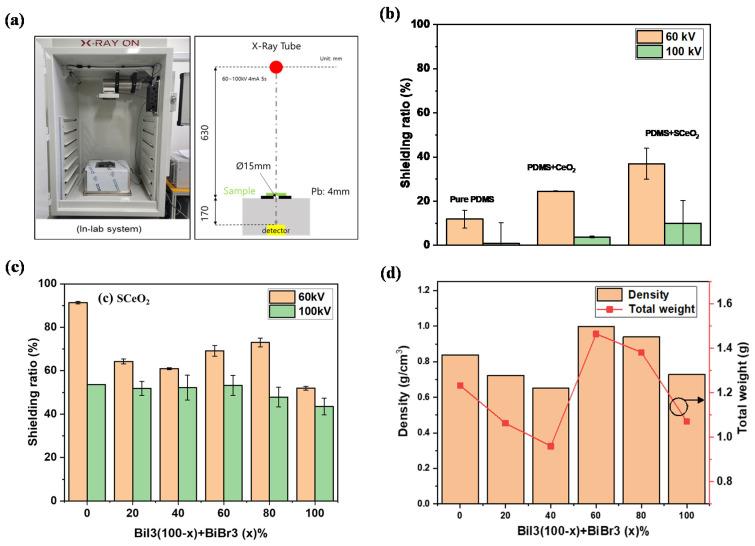
(**a**) Photograph of the in-lab X-ray system for the transmission measurements of coin-shaped PDMS/S-CeO_2_/BiI_3_/BiBr_3_ (**b**) X-ray-shielding ratios of pure PDMS, PDMS/CeO_2_, and PDMS/S-CeO_2_ using 60- and 100-kV X-rays, (**c**) X-ray-shielding ratios of S-CeO_2_/PDMS/BiI_3_/BiBr_3_ using 60- and 100-kV X-rays. (**d**) Density and total weight of the samples.

Based on the successful preparation of the multicomponent PDMS/S-CeO_2_/BiI_3_ composites, we explored the X-ray-shielding performance of each sample, using the X-ray measurement system shown in Figure 6a. First, we compared the X-ray-shielding performance of pure PDMS, PDMS/CeO_2_ sponge, and PDMS/S-CeO_2_ sponge. The pure PDMS exhibited a low shielding performance, below 20%, at tube voltages of 60 and 100 kV. Owing to its low atomic number, low density, and soft and flexible characteristics, pure PDMS is not an effective X-ray-shielding material, allowing X-rays to penetrate the material, rather than be adsorbed or scattered. In contrast, PDMS/CeO_2_ showed an increased shielding performance compared to pure PDMS, owing to the addition of high-*Z* CeO_2_. The shielding ratio was further enhanced for PDMS/S-CeO_2_. Crucially, because sulfated functionalization increases the surface electron density of the metal oxide, there is a greater chance for X-ray photons to interact with the electrons in the composite, lowering the photon energy, and attenuating the X-ray beam [35,36]. Thus, the results confirm that sulfation improved the radiation-shielding performance. However, there are still some regions of the PDMS with a low attenuation, and empty spaces in the sponge structure.

To enhance the shielding performance further, bismuth halides were incorporated into the metal oxide sponge, through it being soaked in a bismuth halide solution. After drying, the bismuth halides covered the PDMS surface, and filled the empty spaces. The X-ray-shielding performance of the PDMS/S-CeO_2_/bismuth halide composite is shown in Figure 6c. Six different compositions of Bi(I_1−*x*_Br*_x_*)_3_ were loaded onto the coin-shaped PDMS/S-CeO_2_ in different weight ratios (*x* = 0, 0.2, 0.4, 0.6, 0.8, and 1). We found that the PDMS/S-CeO_2_/BiI_3_ (*x* = 0) exhibited the best X-ray-shielding ratio of 91.8%, at a tube voltage of 60 kV. In contrast, the PDMS/S-CeO_2_/BiBr_3_ (*x* = 1) exhibited the lowest performance, and there was no benefit from the mixed-halide compositions (Bi(I_1−*x*_Br*_x_*)_3_, *x* = 0.2–0.8) The X-ray-shielding ability is affected by two factors: the attenuation cross-section and electron density. Because BiBr_3_ is smaller, and can form a much denser composite than BiI_3_, it was expected that BiBr_3_ would have a higher-attenuation cross-section. However, BiBr_3_ has a lower electron density than BiI_3_. Considering that both PDMS/S-CeO_2_/BiI_3_ and PDMS/S-CeO_2_/BiBr_3_ already have high-attenuation cross-sections, the effect of the electron density is more crucial to the radiation-shielding performance. Therefore, PDMS/S-CeO_2_/BiI_3_ exhibited a better performance than PDMS/S-CeO_2_/BiBr_3_.

To determine the densities of the composites, we measured the total weight of each sample, and calculated its density. All samples had a coin shape, with a diameter of 25 mm and a thickness of 3 mm, yielding a volume of 1.47 cm^−3^. Because the total weight of the PDMS/S-CeO_2_/BiI_3_ composite is 1.25 g, the calculated density is 0.85 g cm^−3^. Similarly, the densities of the other composites were calculated, and are shown in Figure 6d. All the composites exhibited a low density of less than 1 g cm^−3^, which is beneficial for lightweight X-ray-shielding applications. The comparison of the X-ray-shielding performance with existing materials is shown in Table S2.

## 3. Materials and Methods

### 3.1. Synthesis of Sulfated CeO_2_

Cerium oxide particles (˂5 µm) were purchased from Sigma–Aldrich (St. Louis, MO, USA), and sulfonated as follows. A mixture of sulfuric acid (15 mL, 1 M H_2_SO_4_) and methanol (20 mL) was used to prepare a suspension of nanostructured CeO_2_ (approximately 1 g). The suspension was sonicated at a high intensity for approximately 2 h. To obtain S-CeO_2_, the product was dried for 24 h at 100 °C. Subsequently, the dry S-CeO_2_ product was characterized (Figure S1).

### 3.2. Fabrication of Porous PDMS and PDMS/S-CeO_2_

Bismuth (III) bromide, bismuth (III) iodide, and sulfuric acid (H_2_SO_4_) 99.999% were purchased from Sigma-Aldrich. Polydimethylsiloxane (PDMS) (Sylgard 184 Silicone Elastomer Kit) was purchased from Dow Corning (Midland, MI, USA). The fabrication of porous PDMS was as follows: PDMS, curing agent, and NaCl were mixed in a 1:0.1:1.5 weight ratio. The mixed solution was centrifuged at 8000 rpm for 20 min at room temperature, three times. Then, the excess PDMS was removed, and the sample was heat-treated at 60 °C for 18 h. Then, the solidified PDMS was cut into coin shapes (thickness: 3 mm, diameter: 25 mm). The PDMS coins were immersed in water, and ultrasonicated at 60 °C for 18 h to remove the NaCl inside the PDMS. The volumes of the removed NaCl determine the porosity of the PDMS.

The preparation of porous PDMS/S-CeO_2_ followed almost the same method as that of porous PDMS. A mixture of PDMS, curing agent, S-CeO_2_, and processed NaCl was prepared in a 1:0.1:1.5:2 weight ratio. Then, the mixed solution was centrifuged at 8000 rpm for 20 min at room temperature, three times. Then, the excess PDMS was removed, followed by heat treatment at 60 °C for 18 h. Finally, the PDMS was cut into coin shapes (thickness: 3 mm, diameter: 25 mm), and the NaCl was removed using an ultrasonic cleaner at 60 °C for 18 h, thus yielding the PDMS/S-CeO_2_.

### 3.3. Porous PDMS/BiI_3_/BiBr_3_ Salt Solutions with Different Weight Ratios

BiI_3_ and BiBr_3_ were combined in the weight ratios of 20%, 40%, 60%, and 80%. The two powders were vigorously mixed in a vial, and the ratios are shown in Table 1. Then, the Bi salts were mixed in tetrahydrofuran (THF) (1.0 g salt in 3 mL THF). The porous PDMS/S-CeO_2_ samples were soaked in the Bi salt solution, and dried at 60 °C for 30 min to eliminate THF.

### 3.4. Shielding Ability

The sample and detector were positioned 630 and 800 mm, respectively, away from the X-ray tube producing monochromatic X-rays (Spellman, Precision X-ray Inc. (Madison, CT, USA), X-Rad IR-160, Cabinet X-ray systems USA). The test energy range was set to 60–100 kV, and the tube current was fixed at 4 mA. The dosage accumulation time for each sample was set to 5 s (average of 5 measurements). The porous S-CeO_2_/BiI_3_ composite was analyzed using a transmission densitometer (UniTeko, Seongnam-si, Korea). A porous S-CeO_2_/BiI_3_ composite was used to assess the X-ray-shielding properties of PDMS containing various ratios of BiI_3_/BiBr_3_. The flux (dose) of X-rays can be controlled by adjusting the distance between the X-ray tube and the sample.

$$\mathrm{Shielding}\;\mathrm{Ratio}\left(\%\right)=\frac{X-D}{X}\times 100$$

Here, X is the transmittance without the sample, and D is the transmittance with the sample.

### 3.5. Instrumentation

The crystal structure of pure CeO_2_ and S-CeO_2_ was characterized via powder X-ray diffractometry (PXRD) using Cu-*K*_α_ radiation (30 kV PANalytical/X’Pert3-Powder), in a 2*θ* range of 10° to 80°. Fourier transform infrared (FT-IR) spectra were obtained using a JASCO/FT-4100 spectrometer. X-ray photoelectron spectroscopy (XPS, KRATOS Analytical Ltd. (Stretford, UK)/AXIS SUPRA) was measured using a monochromatic Al *K*_α_ source, with a spot size of 400 µm and a pass energy of 40 eV. The morphology of the synthesized S-CeO_2_ was analyzed using high-resolution transmission electron microscopy (HR-TEM, JEM-F200, JEOL Ltd., Tokyo, Japan), and selected-area electron diffraction (SAED), at an acceleration voltage of 200 kV. In addition, energy-dispersive X-ray spectroscopy (EDX) measurements and mapping were carried out. Field-emission scanning electron microscopy (FE-SEM, MIRA3 TESCAN, TESCAN KOREA, Seoul, South Korea) and energy-dispersive X-ray spectroscopy (EDX) were performed, to produce EDX spectra and elemental maps. The X-ray-shielding performance was measured using an X-ray instrument (Spellman, Precision X-ray Inc., X-Rad IR-160, Cabinet X-ray systems, USA) producing X-rays at the tube voltages of 60 and 100 kV, and a current of 4 mA, for 5 s.

## 4. Conclusions

We have successfully demonstrated efficient and lightweight X-ray-shielding materials based on S-CeO_2_ and bismuth halides for the first time. The sulfation of the metal oxides enhanced their X-ray-shielding ability, as confirmed by structural, chemical, and spectroscopic analyses. The integration of the doped metal oxide as a porous sponge, and bismuth halide as a filler resulted in uniform, compact, and intimate composites with a high-attenuation cross-section. The thin (approximately 3 mm) and lightweight (0.85 g·cm^−3^) composites achieved an excellent X-ray-shielding rate of approximately 92% at 60 kV, which is among the highest values reported for non-heavy-metal shielding materials. This multicomponent lightweight PDMS/S-CeO_2_/BiI_3_ composite has potential applications in wearable X-ray-shielding garments, medical imaging, and nuclear power plant safety.

## Figures and Tables

**Table 1:** The composition of bismuth halide mixtures used to prepare the PDMS/S-CeO_2_/Bi(I_1−*x*_Br*_x_*)_3_ composites. A0, A2, A4, A6, A8, and A10 represent the samples with different ratios of BiBr_3_ and BiI_3_. Powder A and powder B are BiBr_3_ and BiI_3_, respectively. The mass of each powder used to make 1 g of bismuth halide mixture is shown in grams.

Sample Name	A0	A2	A4	A6	A8	A10
Powder A (g) BiBr_3_	0	0.2	0.4	0.6	0.8	1
Powder B (g) BiI_3_	1	0.8	0.6	0.4	0.2	0

## Data Availability

Not applicable.

## Supplementary Materials

The following supporting information can be downloaded at: https://www.mdpi.com/article/10.3390/molecules28166045/s1, Figure S1: Additional data for sample preparation; Figure S2: EDX of S-CeO_2_; Figure S3: SEM images of PDMS; Figure S4: Elemental mapping of PDMS; Figure S5: SEM images of PDMS/S-CeO_2_; Figure S6: Elemental mapping of PDMS/S-CeO_2_; Figure S7: SEM images of PDMS/S-CeO_2_/BiI_3_; Figure S8: Elemental mapping of PDMS/S-CeO_2_/BiI_3_; Table S1: Quantitative data on the chemical composition; Table S2: Comparison of X-ray-shielding performances. References [37,38,39,40,41,42,43,44] are cited in the Supplementary Materials.

## Sample Availability

Samples of the compounds mentioned in this study are available from the authors.
