# Supplementary material for: Multicomponent X-ray Shielding Using Sulfated Cerium Oxide and Bismuth Halide Composites
Source: Molecules. 2023 Aug 14;28(16):6045. doi: 10.3390/molecules28166045 (PMC10457930; doi:10.3390/molecules28166045)
Supplement: Supplementary file 1 [file molecules-28-06045-s001.zip › molecules-2540550-supplementary.pdf]

Supporting Information

Multicomponent X-ray Shielding using Sulfated Cerium Oxide and Bismuth Halide Composites

Shanmugam Mahalingam[1], Dae Seong Kwon[1], Seok Gyu Kang[1], and Junghwan Kim[1,2*]

[1]Department of Materials System Engineering, Pukyong National University, Busan 48513, Republic of Korea

[2]Institute of Energy Transport and Fusion Research, Pukyong National University, Busan 48513, Republic of Korea

This supplementary information includes:

A. Scheme for the preparation of Sulfated CeO_2 and X-ray shielding performance

B. EDX of Sulfated CeO_2

C. Morphology of coin shape PDMS

D. Elemental mapping of PDMS with EDX

E. Morphology of coin shape PDMS/S-CeO_2

F. Elemental mapping of coin shape PDMS/S-CeO_2 with EDX

G. Morphology of coin shape PDMS/S-CeO_2/BiI_3

H. Elemental mapping of coin shape PDMS/S-CeO_2/BiI_3 with EDX

I. Quantitative data on the chemical composition of the shielding materials

J. Comparison of X-ray shielding performances.

Figures S1 to S8

Table S1 to S2

A. Scheme for the preparation of Sulfated CeO_2 and X-ray shielding performance

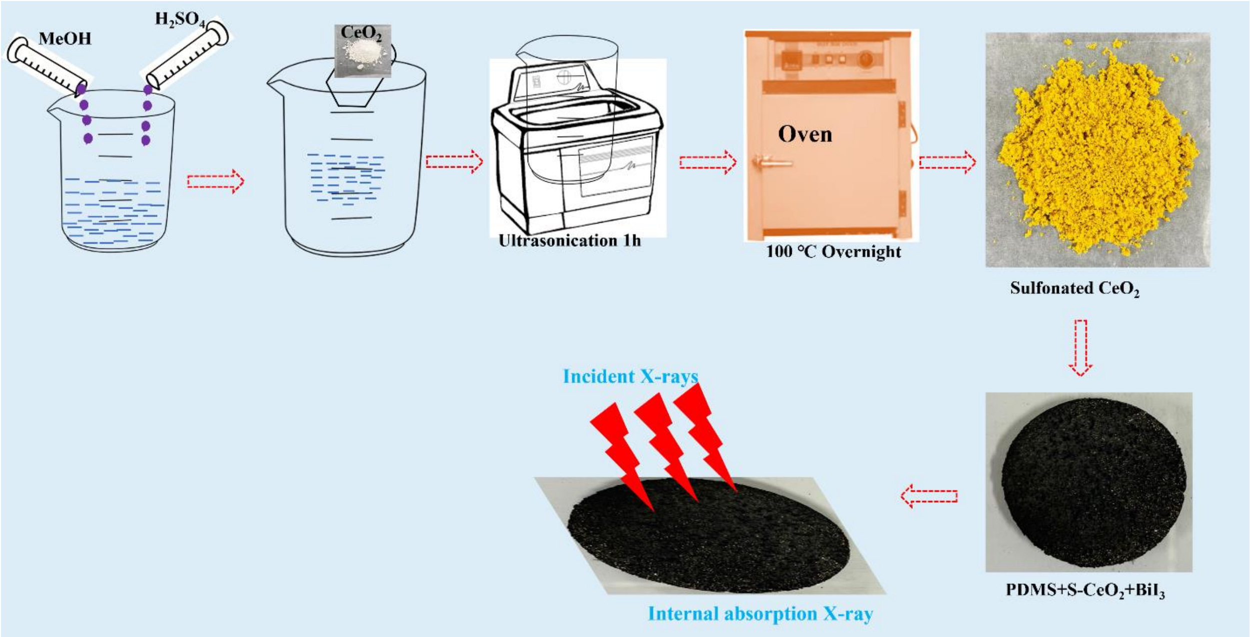

Figure S1. Scheme for the preparation of Sulfated CeO_2 and X-ray shielding performance.

B. EDX of Sulfated CeO_2

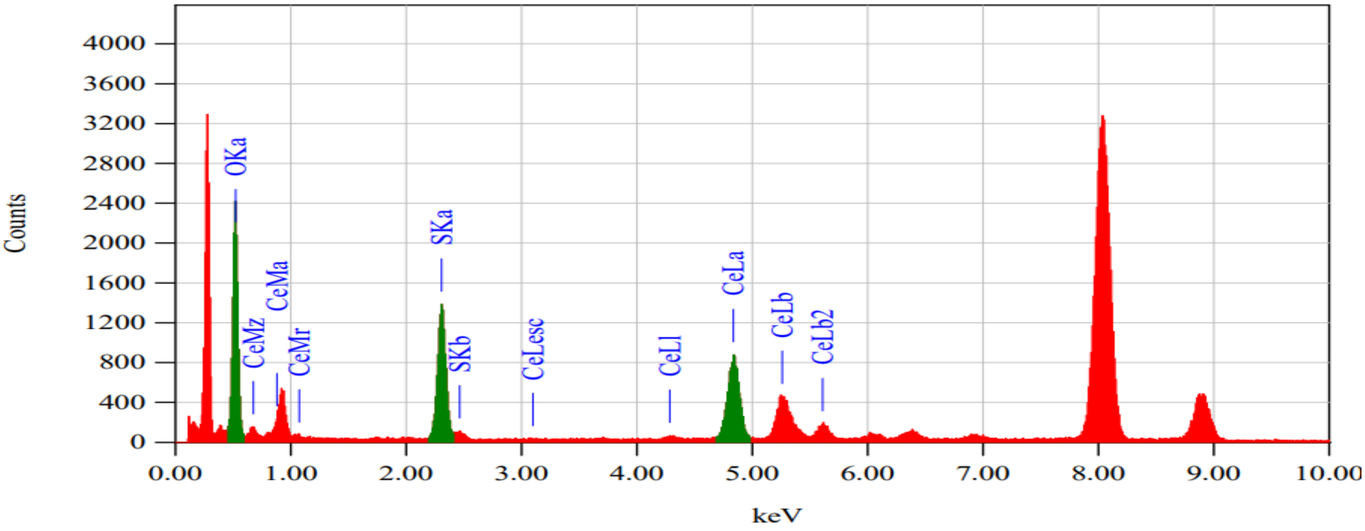

Figure S2. EDX of Sulfated CeO_2

C. Morphology of coin shape PDMS

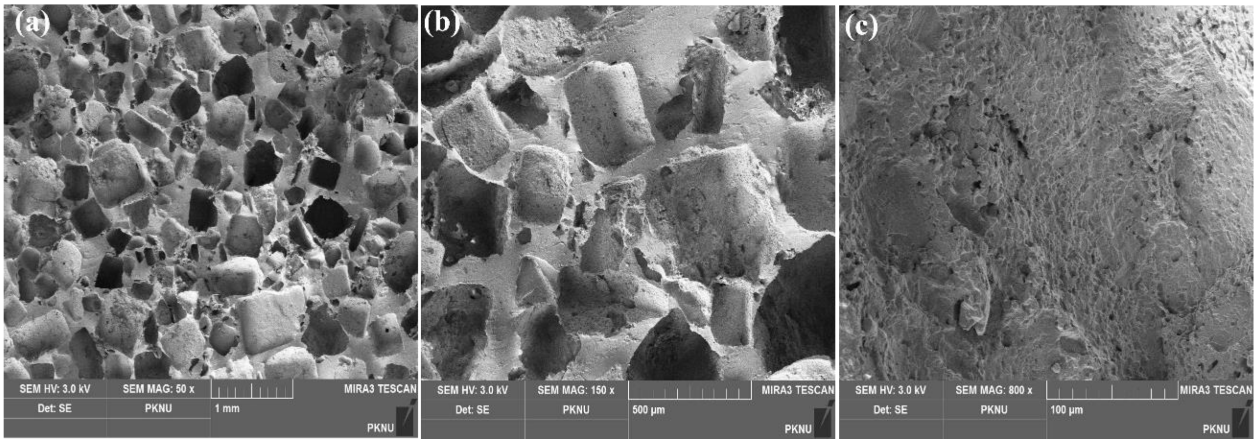

Figure S3. SEM of pure PDMS

D. Elemental mapping of PDMS with EDX

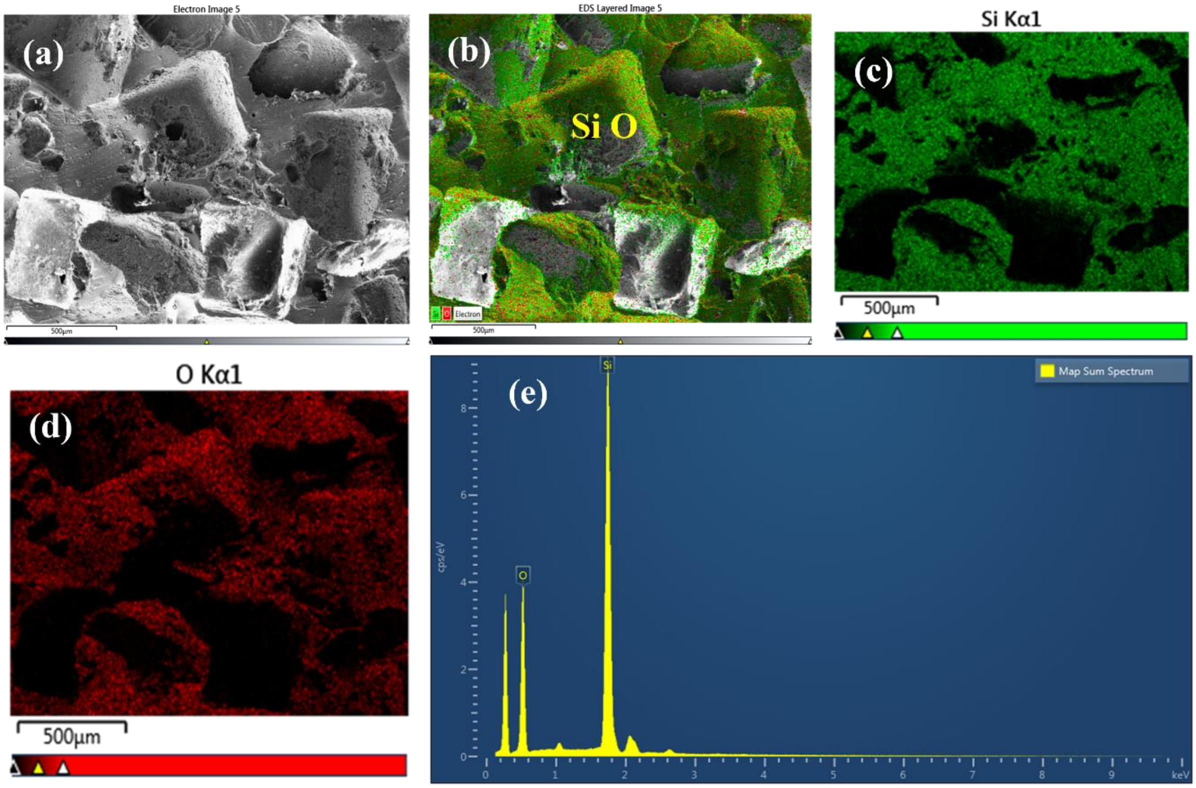

Figure S4. Elemental mapping with EDX of Pure PDMS

E. Morphology of coin shape PDMS/S-CeO_2

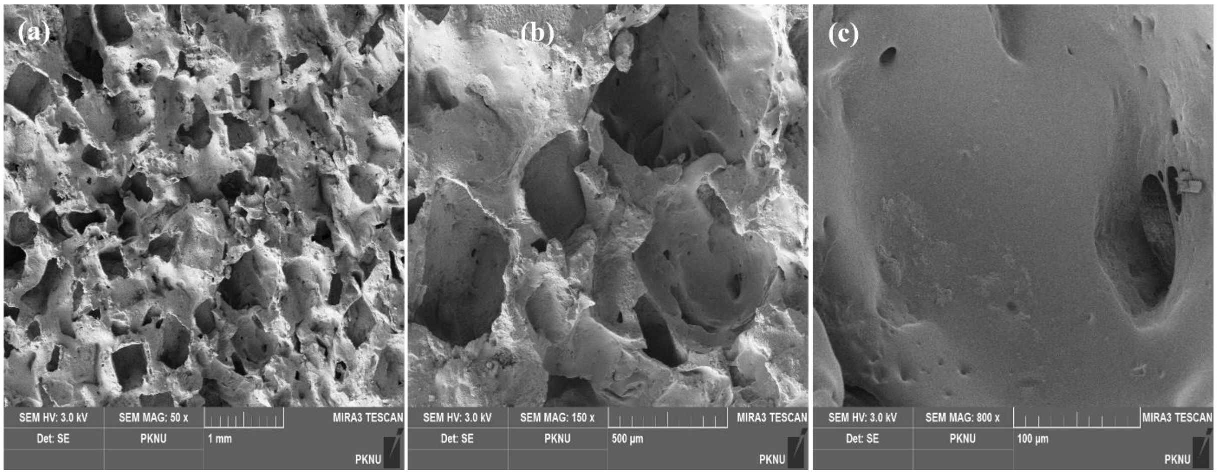

Figure S5. SEM of PDMS/S-CeO_2

F. Elemental mapping of coin shape PDMS/S-CeO_2 with EDX

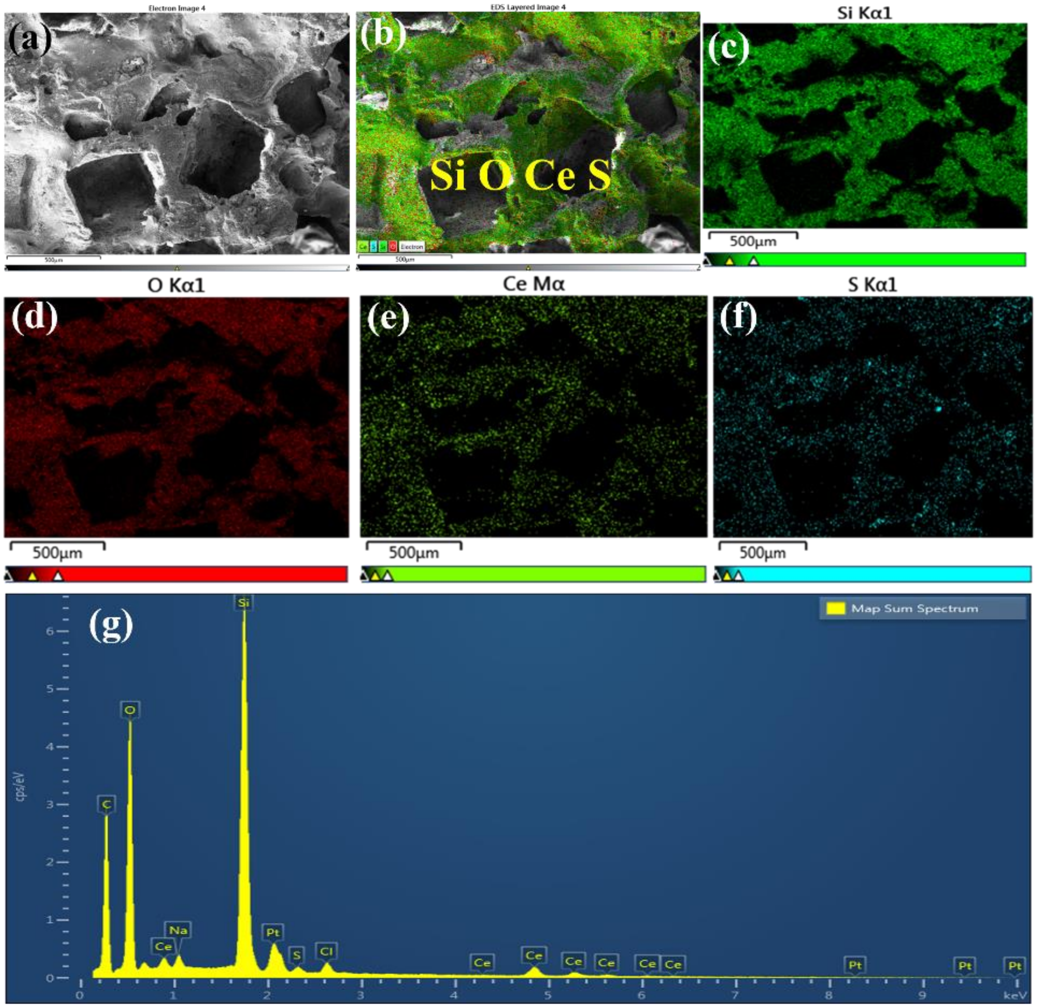

Figure S6. Elemental mapping with EDX of PDMS/S-CeO_2

G. Morphology of coin shape PDMS/S-CeO_2/BiI_3

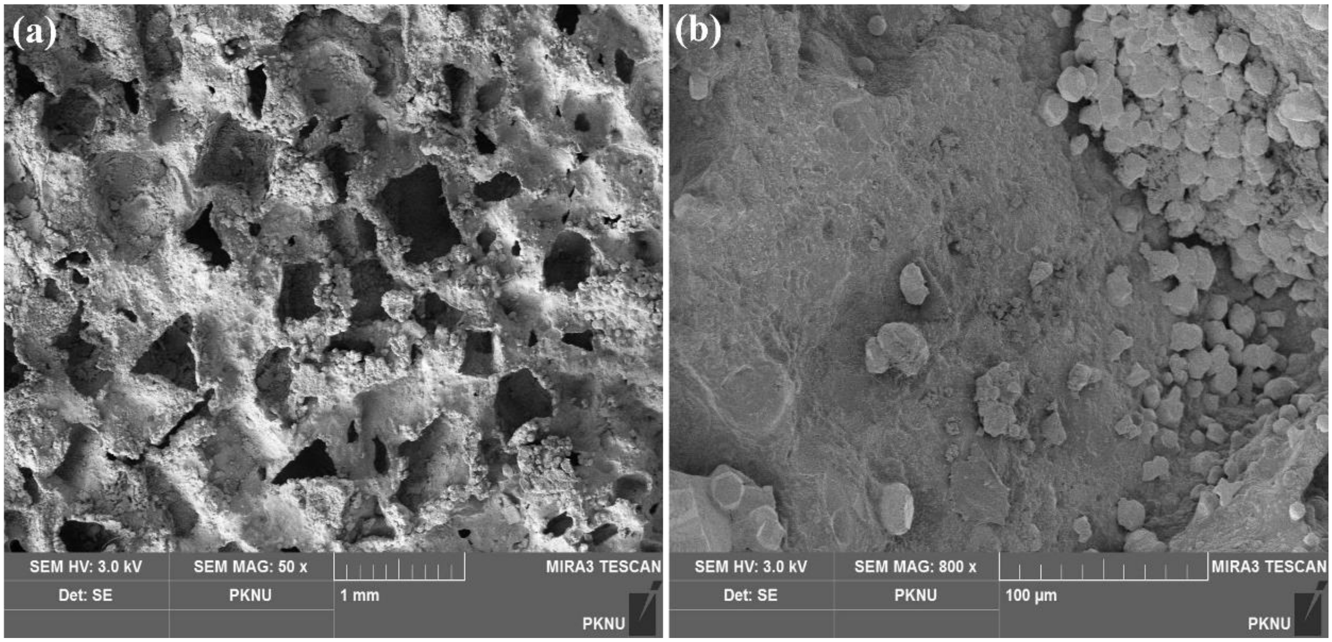

Figure S7. SEM of PDMS/S-CeO_2/BiI_3

H. Elemental mapping of coin shape PDMS/S-CeO_2/BiI_3 with EDX

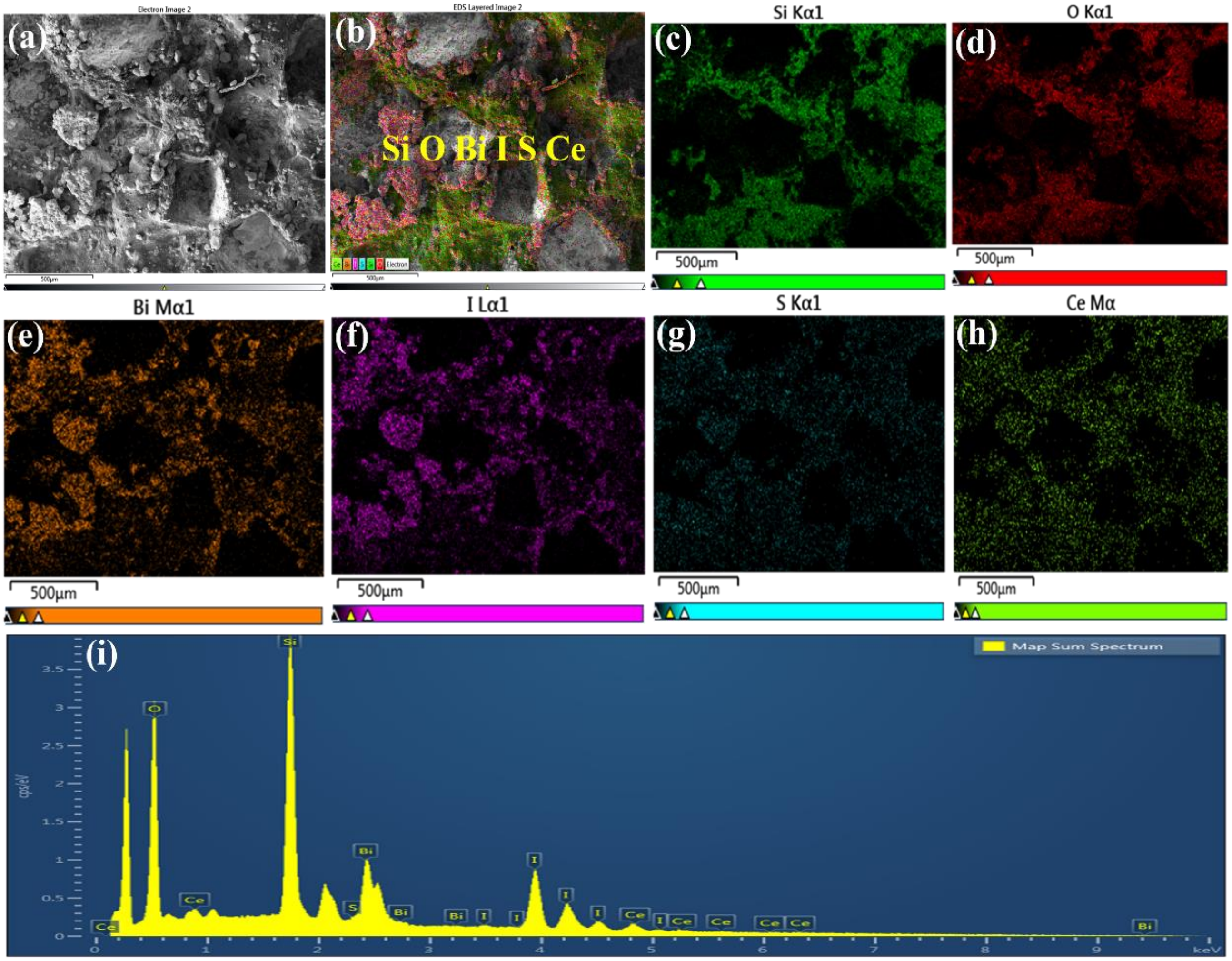

Figure S8. Elemental mapping with EDX of PDMS/SCeO_2/BiI_3

I. Quantitative data on the chemical composition of the shielding materials

Table S1. EDX Quantitative data on the chemical composition of the PDMS/S-CeO_2/BiI_3 shielding material.

No	Element	Wt%	Atomic %
1	**O**	12.29	40.50
2	**Si**	17.65	33.14
3	**S**	0.72	1.19
4	**I**	42.76	17.77
5	**Ce**	5.60	2.11
6	**Bi**	20.97	5.29
	Total:	100.00	100.00

J. Comparison of X-ray shielding performances.

Table S2. Comparison of X-ray shielding performances

No	X-ray shielding material	Voltage (kV)	X-ray shielding performance (%)	References
1.	$BaSO_4$/cellulose nanocomposite membranes	50 kV	81.70%	[38]
2.	Bi@Bi-La Natural Leather Composites	20-120keV	65-100%	[39]
3.	Bi_2O_3/PVA composite	100 kVp	80%	[40]
4.	Bi/Silicone rubber/cotton fabric	100kV	90%	[41]
5.	Bi_2O_3/polyvinyl chloride (PVC)/nylon fabrics	80kV	83.00%	[42]
6.	PVA/ gelatin/ $BaCO_3$/wood composite	77kV	53.18	[43]
7.	Bi/Ce-natural leather composite	40 keV	~100%	[44]
8.	PANi matrix embedded with WO_3 and GO-TBT	120 keV	98%	[45]
9.	Porous PDMS/S-CeO_2/ BiI_3	60kV	~92%	This work

REFERENCES

[38] Jiang, X.; Zhu, X.; Chang, C.; Liu, S.; Luo, X. X-ray shielding structural and properties design for the porous transparent $BaSO_4$/cellulose nanocomposite membranes, Int. Jour. of Biolog. Macromol. **2019,** 13, 9793–800.

[39] Li, Q.; Zhong, R.; Xiao, X.; Liao, J.; Liao, X.; Shi, B. Lightweight and Flexible Bi@Bi-La Natural Leather Composites with Superb X ray Radiation Shielding Performance and Low Secondary Radiation, ACS Appl. Mater. Interfaces. **2020,** 12, 54117–54126.

[40] Kaewpirom, S.; Chousangsuntorn, K.; Boonsang, S. Evaluation of Micro- and Nano-Bismuth (III) Oxide Coated Fabric for Environmentally Friendly X Ray Shielding Materials, ACS Omega. **2022,**7, 28248–28257.

[41] Aral, N.; Banu Nergis, F.; Candan, C. An alternative X-ray shielding material based on coated textiles, Textile Research Journal. **2016,** 86, 803-811.

[42] Maghrabi, H.A.; Vijayan, A.; Deb, P.; Wang, L. Bismuth oxide-coated fabrics for X-ray shielding, Textile Research Journal. **2016,** 86, 649–658.

[43] Muhammad, N.A.; Armynah, B.; Tahir, D. High transparent wood composite for effective X-ray shielding applications, Mat. Resea. Bull. **2022,** 154, 111930.

[44] Li, Q.; Wang, Y.; Xiao, X.; Zhong, R.; Liao, J.; Guo, J.; Liao, X.; Shi, B. Research on X-ray shielding performance of wearable Bi/Ce-natural leather composite materials, Jour. Of. Hazar. Mat. **2020,** 398, 122943.

[45] Zarei, M.; Sina, S.; Hashemi, S.A. Superior X-ray radiation shielding of biocompatible platform based on reinforced polyaniline by decorated graphene oxide, with interconnected tungsten–bismuth–tin complex, Rad. Phys, and Chem. **2021,** 188,109588.
